# Application of X-ray powder diffraction and differential scanning calorimetry for identification of counterfeit drugs

**DOI:** 10.1007/s00706-018-2193-z

**Published:** 2018-04-10

**Authors:** Izabela Jendrzejewska, Paweł Zajdel, Ewa Pietrasik, Zoja Barsova, Tomasz Goryczka

**Affiliations:** 1 Institute of Chemistry, University of Silesia, Katowice, Poland; 2 Institute of Physics, University of Silesia, Katowice, Poland; 3 Institute of Materials Science, University of Silesia, Chorzów, Poland

**Keywords:** Solid state, Drug research, Differential scanning calorimetry, X-ray investigation

## Abstract

**Abstract:**

X-ray analysis confirmed that in all investigated samples, the active API (acetylsalicylic acid and ascorbic acid) was present. The values of the interplanar distance *d*_*hkl*_ for the studied samples are in good accordance with those presented in the ICDD database. The intensities of the diffraction lines depend on the content of the component in the tested preparation. Therefore, different intensities of lines for the APIs were observed in the obtained diffraction patterns. Thermal analysis of the studied substances showed that during the thermal analysis, the following phenomena might occur: dehydration and (or) melting, crystalline transformation. Moreover, it was found that the chemical structure of the studied compounds affects the process of their thermal decomposition. The data obtained during these investigations can be useful in quick tests of physicochemical discrepancies and abnormalities between potential components of pharmaceutical preparations. The evidence for the interaction can be obtained by comparing DSC and TG curves of the drug and the excipient, as well as those of their physical mixtures. For this reason, the study of characteristics of thermal decomposition of drugs and excipients is necessary. Based on the above investigations, it may be stated that a combination of two methods: XRPD and DSC can be used to distinguish the original drugs from counterfeit products, e.g., by checking for the presence of the correct API or by a comparison of the drugs fingerprint.

**Graphical Abstract:**

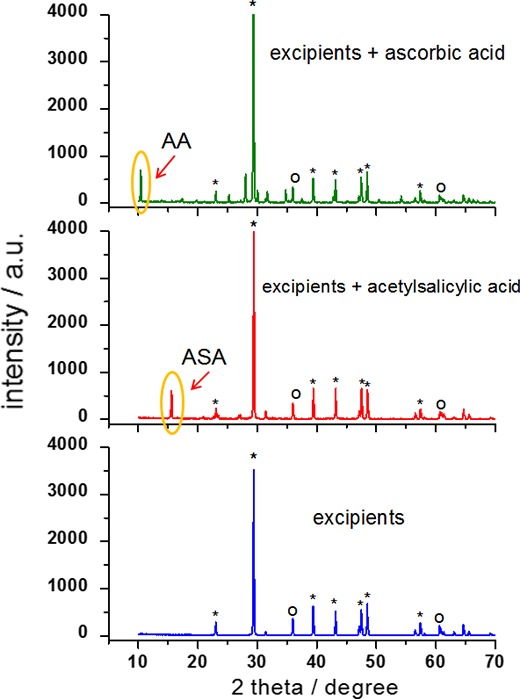

## Introduction

A significant increase in the availability of drugs, especially the possibility to purchase medical preparations in shops, at petrol stations, or online, creates the possibility for introducing counterfeit drugs, which can contain inappropriate substances, abnormal amounts of active substances or significant amount of impurities. The World Health Organization estimates that approx. 50% of the drugs for sale on the internet are falsified [1]. In addition, a very large number of advertisements, lower prices for these drugs, and emerging “discounts” for customers further encourage the purchase.

We can find the counterfeit drugs in brand and generic name pharmaceuticals, as well as in developing and developed countries. It has been observed that specific groups of medical preparations are counterfeited much more often than others. These groups include antibiotics, antihistamines, anti-malarials, hormones, steroids, and others [2].

Counterfeit pharmaceuticals may threaten the health and life, and for this reason, it is important to monitor and investigate the pharmaceutical materials. Since the most of drugs are solid crystalline compounds, we can use common techniques such as diffraction (X-ray, neutron, electron), thermal analysis (DSC, DTA, TG…), spectroscopic (IR, NIR, Raman), micromeritics (surface area, particle size, iGC, vapor sorption), microscopy (optical, SEM, TEM, STM, AFM), and their combinations. A combination of two or more test methods allows for confirming the authenticity and composition of a drug. In our investigations, a combination of XRPD (X-ray powder diffraction) and DSC (differential scanning calorimetry) methods was used to check the presence of API (active pharmaceutical ingredient) in selected drugs and dietary supplements,.

**Fig. 1:**
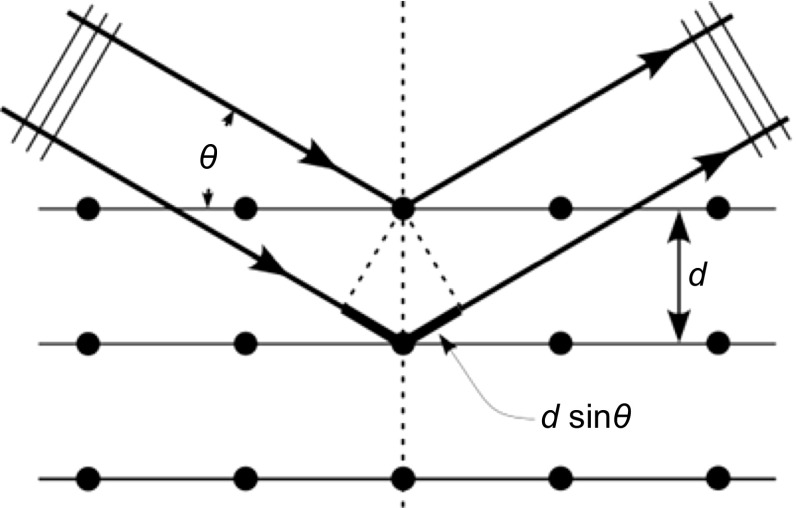
Bragg’s law and a graphical representation of incident X-rays diffracting from atoms within different crystalline layers (https://en.wikipedia.org/wiki/X-ray_crystallography#/media/File:Bragg_diffraction_2.svg. Accessed 10 Sept 2017)

A necessary condition for application of X-ray diffraction for substance investigations is constituted by crystallinity of the investigated substance. This means that only a substance having crystalline structure gives satisfactory results. Amorphous (non-crystalline) substances are not suitable for this type of analysis. Crystalline materials are characterized by ordered periodic arrangements of atoms. Atoms in a crystal constitute a periodic array of coherent scatterers and thus can diffract electromagnetic radiation. X-ray diffraction is used to obtain structural information about crystalline solids. The diffraction occurs when the wavelength is comparable to atomic spacings—the radiation is scattered by electrons of a crystalline system then. For a crystalline solid, the waves are scattered by lattice planes separated by the interplanar distance *d*_*hkl*_. When the scattered waves interfere constructively, they remain in phase since the difference between the path lengths of the two waves is equal to an integer multiple of the wavelength. The path difference between two waves undergoing interference is given by Bragg’s law [3]:

1 $$d_{hkl} = \frac{n\lambda }{{2{ \sin \theta}}},$$

where *n* is the diffraction order, *λ* is the X-ray wavelength, *θ* is the angle of deflection, and *hkl* are the Miller’s indices. The value of *d*_*hkl*_ is an intrinsic, instrument-independent, material property (Fig. 1).

Based on the X-ray examination, a certain amount of information about the studied material may be obtained. In qualitative analysis, the position of the reflection (deflection angle, interplanar distance *d*_*hkl*_), which provides information about the sample’s qualitative composition, is used.

Identification of crystalline substance using the X-ray diffraction (XRD) method consists in determining the interplanar distance *d*_*hkl*_ for the investigated substance, as well as the intensity of the deflection from these X-ray planes, and comparing their values with the data contained in the diffraction data library. The diffraction dataset for crystalline substances has been developed by an international institution International Centre for Diffraction Data (ICDD). In our study, we use ICDD database called PDF-2 (release 2008). This database contains more than 200 000 crystalline substances, which are helpful for identification of the investigated material [4].

Thermal analysis is a branch of materials science where the properties of materials are studied as they change with temperature. Thermogravimetry (TG) is the measurement of the mass of a sample as the temperature increases. This method is useful for determining sample purity and water, carbonate, and organic contents; and for studying thermal decomposition reactions. Differential scanning calorimetry (DSC) independently measures the rate of heat flow to a sample and to a standard that are at the same temperature. This method is used for determining such properties as: melting/crystallization behavior, solid–solid reactions, degree of crystallinity, glass transitions, decomposition behavior, purity determination, and specific heat.

## Results and discussion

Our interests have focused on popular and easily accessible drugs. For this study, the selected over-the-counter medicines and dietary supplements containing acetylsalicylic acid (ASA) and ascorbic acid were chosen.

Aspirin and vitamin C are one of the most popular general purpose drugs, which make them perfect targets for mass and cheap forgeries. They are commonly used with other medicines, which does not immediately raise suspicions in case they are not working. This procedure is not only limited to substances in their pure form but is widely spread among dietary supplements containing these active ingredients. In 2013, French customs officers have seized 1.2 million doses of counterfeit Aspirin from China. It was found to contain mostly glucose without any active ingredients. It was the biggest haul of fake medicines ever in France and the EU [5]. Similarly, dietary supplements containing vitamin C was fake. In the USA, the Food and Drug Administration (FDA) advised the public against the use of the product Tatiomax, in which one of the components is vitamin C [6].

For this work, we selected examples of popular over-the-counter drugs containing acetylsalicylic and ascorbic acids. As reference standards, we used acetylsalicylic acid obtained in our laboratory and ascorbic acid purchased from Sigma-Aldrich. All materials are listed in Tables 1 and 2.

**Table 1:** Investigated drugs containing acetylsalicylic acid

No.	Drug	Amount of acetylsalicylic acid (in 1 tablet)
1	Acard	75 or 150 mg (Polfa)
2	Aspirin Bayer	500 mg (Bayer GmbH)
3	Aspirin C	400 mg 240 mg of ascorbic acid (Bayer GmbH)
4	Aspirin Effect	500 mg (Bayer GmbH)
5	Coffepirine	450 mg 50 mg of caffeine (Medicofarma)
6	Pure acetylsalicylic acid	Chemical reagent (Sigma-Aldrich)

**Table 2:** Investigated drugs containing ascorbic acid

No.	Drug		Amount of ascorbic acid (in 1 tablet)
1	Rutinoscorbin	100 mg 25 mg of Rutosidum trihydricum (GlaxoSmithKline)	
2	Vitaminum C	200 mg (Alofarm)	
3	Gripex HotActive	100 mg 1000 mg of paracetamolum 12.2 mg of Phenylephrini hydrochloridum (USP Zdrowie)	
4	Pyramidon	300 mg 500 mg of acetylsalicylic acid 200 mg of calcium (Adamed)	
5	Vitaminum C	200 mg (Apteo)	
6	Pure ascorbic acid	Chemical reagent (Sigma-Aldrich)	

The materials were characterized using the XRPD and DSC/TG methods. To check the standards, the data obtained from X-ray diffraction were compared against ICDD cards: 00-001-0182 for acetylsalicylic acid and 00-022-1536 for ascorbic acid (Tables 3 and 4) [7].

**Table 3:** The comparison of data for acetylsalicylic acid obtained in laboratory with data from ICDD database (PDF 00-001-0182)

No. of diffraction line*	2*θ* from ICDD/°	2*θ* from diffraction pattern/°	*d*_*hkl*_ from ICDD/Å	*d*_*hkl*_ calculated/Å	*h*	*k*	*l*
1	7.5497	7.7842	11.70	11.50	1	0	0
2	15.5881	15.6435	5.70	5.71	0	0	2
3	20.6874	20.6483	4.29	4.30	− 3	1	1
4	22.6647	22.6242	3.92	3.93	− 5	1	1
5	26.9968	26.9551	3.30	3.28	0	4	0

*The first 5 lines

**Table 4:** The comparison of data for ascorbic acid purchased as chemical reagent with data from ICDD database (PDF 00-022-1536)

No. of diffraction line*	2*θ* from ICDD/°	2*θ* from diffraction pattern/°	*d*_*hkl*_ from ICDD/Å	*d*_*hkl*_ calculated/Å	*h*	*k*	*l*
1	10.2895	10.4485	8.59	8.46	2	0	0
2	17.4774	17.4213	5.07	5.09	2	1	0
3	19.8457	19.8210	4.47	4.48	0	1	1
4	28.0720	28.0541	3.18	3.18	0	0	2
5	30.0634	30.0167	2.97	2.97	− 3	0	2

*The strongest lines

To show the diffraction pattern for excipients only and for API together with excipients, we prepared a blank test. As APIs, pure acetylsalicylic acid and ascorbic acid were used. The excipients were in the form of a tablet which included CaCO_3_, Mg stearate, and talc, and blank samples contained 150 mg API. Each sample was measured using XRPD method in the range of angle 2*θ* = 10–135°. This experiment allowed for showing the difference between the sample with API and without API, and might be an indication in investigations of counterfeit drugs.

**Fig. 2:**
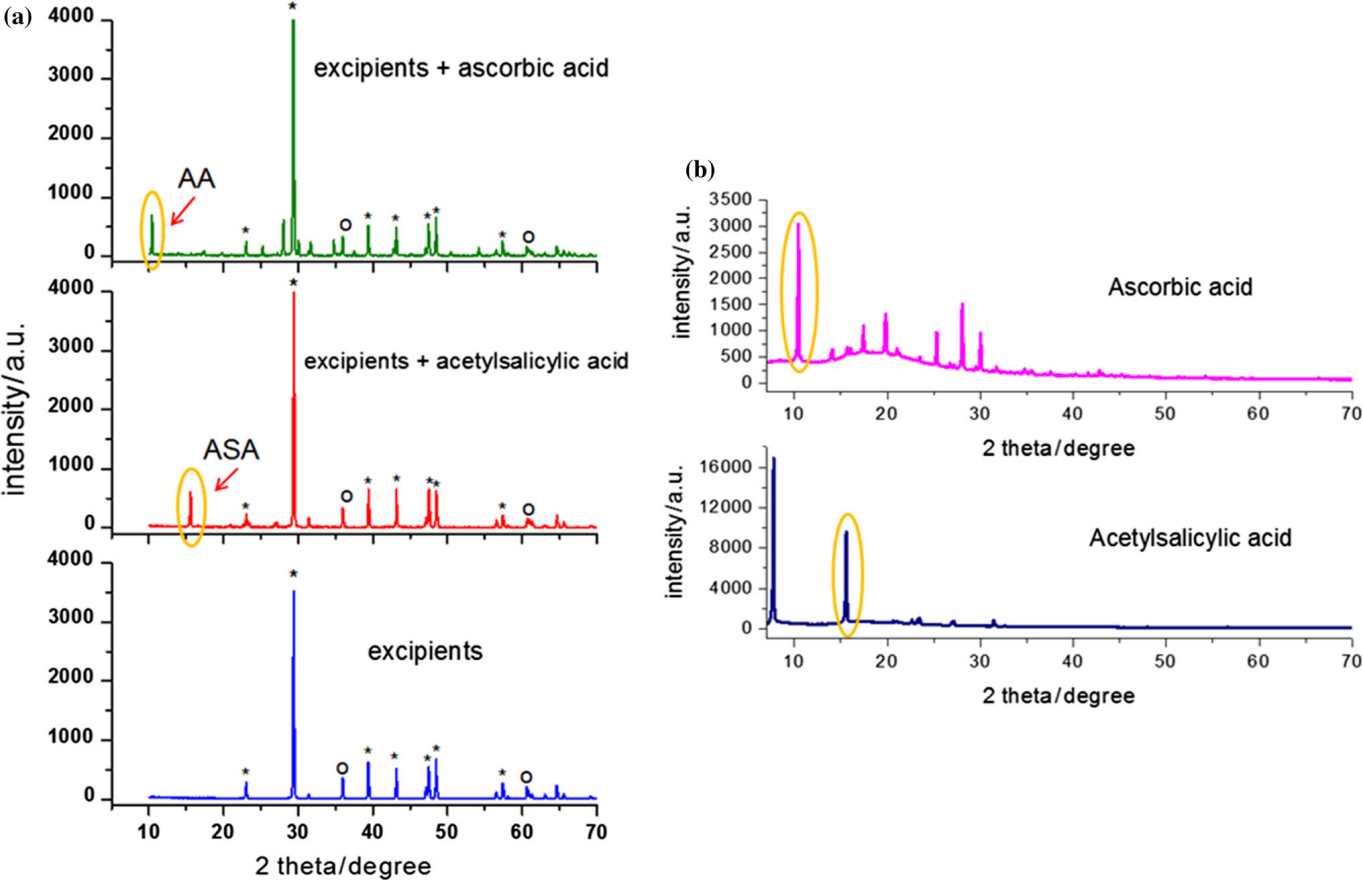
Diffraction patterns for blank test samples: **a** pure excipients and excipients with API, **b** pure APIs, which were used as standards

Diffraction patterns for pure acetylsalicylic acid and ascorbic acid, which are presented in Fig. 2b, were used as standards, whereas for identification of remaining ingredients (Fig. 2a), we used the data available in the ICDD database: PDF cards No. 00-001-0837 for CaCO_3_, No. 00-003-0887 for talc, and No. 00-054-1972 for magnesium stearate. Thanks to these data, the diffraction lines from excipients and APIs were identified. The strongest diffraction line according to diffraction data for magnesium stearate is for angle of 2*θ* = 5.4177°. Since the measurements for these three samples started from 10°, this line is absent on our patterns.

**Fig. 3:**
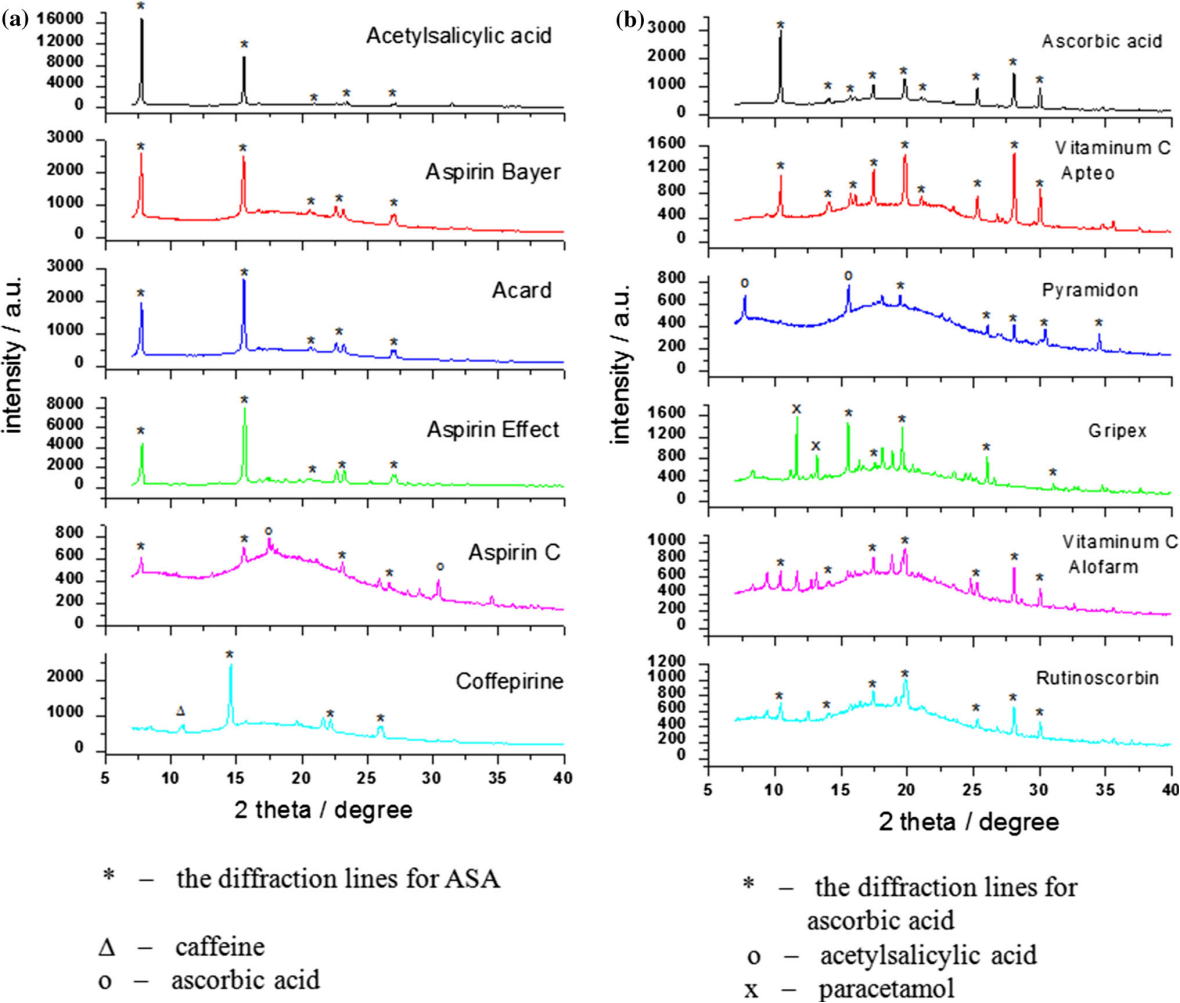
Diffraction patterns for selected drugs containing acetylsalicylic acid (**a**) and ascorbic acid (**b**)

The XPRD analysis confirmed that in all investigated drugs, the active APIs (acetylsalicylic acid and ascorbic acid) were present, as well as the diffraction lines of other APIs were observed in their diffraction patterns (Fig. 3).

For some medicines, the diffraction lines originating from other ingredients included in the composition of the drug, where observed. Next to the main ingredients, we observed the presence of caffeine and paracetamol. Similarly, the data for other ingredients have been taken from the ICDD database, too [7].

A separate treatment was dedicated to detection of magnesium stearate, which is often used as a lubricant in the pharmaceutical industry. We did not observe any diffraction lines characteristic for this compound in all commercial samples, which does not exclude its presence as an amorphous addition. However, a chemical incompatibility between aspirin and magnesium stearate can lead to appearance of a number of potentially undesirable products, such as salicylic acid [8]. Therefore, in all drugs containing acetylsalicylic acid, the diffraction line characteristics for salicylic acid were not observed (the strongest diffraction line for salicylic acid is for angle of 2*θ* = 25.2806°, PDF card 00-001-0558).

**Fig. 4:**
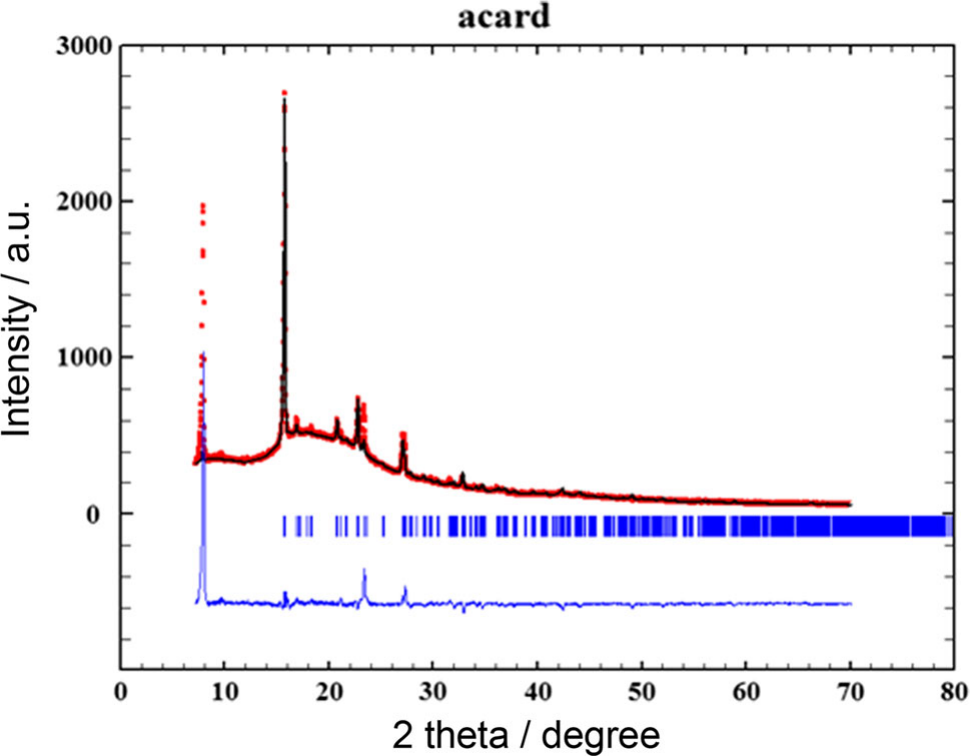
Result of the Rietveld refinement

X-ray diffraction pattern for Acard was fitted as an example using Rietveld method using model structure of I type of aspirin (COD 7050897) with space group P2_1_/c. Refinement of the X-ray diffraction pattern was performed using Fullprof programme [9]. Result of this refinement is shown on Fig. 4. It has to be noted that full Rietveld refinement is not routinely used for phase identification purposes and requires high purity samples. Additionally, even for a pure acetylsalicylic acid, which is low Z (13 non hydrogen C, O and 8 H atoms), low symmetry (monoclinic) material, Rietveld refinement requires a high-q, high statistics data available only at synchrotron sources. Phase ID is usually performed in short, low angle range of pattern, therefore, it is not suitable for this purpose.

In any case, our refinement with fixed atomic coordinates gave in relatively low values of Rietveld factors: *R*_p_ = 3.60, *R*_wp_ = 5.59, and *R*_exp_ = 1.60, which indicates generally good agreement between the model and the sample. It has to be noted that the sample had to be corrected for preferred orientation along (100) and the obtained structural results due to mounting method.

The diffraction patterns are best reported using the interplanar distance *d*_*hkl*_ rather than the value of 2*θ* angle. The peak position at 2*θ* angle depends on instrumental characteristic such a wavelength. The value of *d*_*hkl*_ is a characteristic material property, which is used for identification of the substance. Therefore, for each investigated sample, the calculations of *d*_*hkl*_ values were done. The value of 2*θ* angle for each diffraction line was drawn from the diffraction pattern. Then, the Bragg’s Law was used to convert the observed 2*θ angle* position to *d*_*hkl*_.

Next, the calculated interplanar distances *d*_*hkl*_ were compared with the *d*_*hkl*_ values from the ICDD database. The calculated values of *d*_*hkl*_ are in a good accordance with those presented in the ICDD database (see Tables 5 and 6).

**Table 5:** The comparison of data obtained for Aspirin Bayer with ICDD database (PDF 00-012-0850)

No. of diffraction line	2*θ* from ICDD/°	2*θ* from diffraction pattern/°	*d*_*hkl*_ from ICDD/Å	*d*_*hkl*_ calculated/Å	*h*	*k*	*l*
1	7.5497	7.6842	11.70	11.60	1	0	0
2	15.5881	15.6435	5.70	5.71	0	0	2
3	16.7453	16.8030	5.29	5.27	− 1	0	2
4	20.6387	20.6035	4.30	4.30	0	1	2
5	22.6063	22.5871	3.92	3.93	2	1	1
6	23.2046	23.1937	3.83	3.83	2	0	2
7	26.9137	26.9451	3.31	3.30	− 3	0	2
8	27.0804	27.1144	3.29	3.29	3	1	0
9	31.3837	31.3617	2.84	2.85	0	2	2
10	32.6057	32.6184	2.74	2.74	3	1	2

**Table 6:** The comparison of data obtained for Vitamin C (Apteo) with ICDD database (PDF 00-022-1536)

No. of diffraction line	2*θ* from ICDD/°	2*θ* from diffraction pattern/°	*d*_*hkl*_ from ICDD/Å	*d*_*hkl*_ calculated/Å	*h*	*k*	*l*
1	10.2895	10.4485	8.59	8.46	2	0	0
2	14.1588	14.1223	6.25	6.26	− 1	0	1
3	15.7274	15.7368	5.63	5.63	1	0	1
4	17.4774	17.4455	5.07	5.09	2	1	0
5	19.8457	19.7949	4.47	4.48	0	1	1
6	21.3933	21.3669	4.15	4.16	− 2	1	1
7	25.078	25.2968	3.53	3.52	4	1	0
8	28.0720	28.0649	3.18	3.18	0	0	2
9	29.4744	29.5429	3.028	3.021	1	0	2
10	30.0634	30.0469	2.97	2.97	− 3	0	2

According to [10] if peak shifts within a suspect product XRD spectrum are greater than 0.2° for a given 2*θ* diffraction angle compared to the authentic XRD spectrum, then the product meets the criteria for a counterfeit product.

**Fig. 5:**
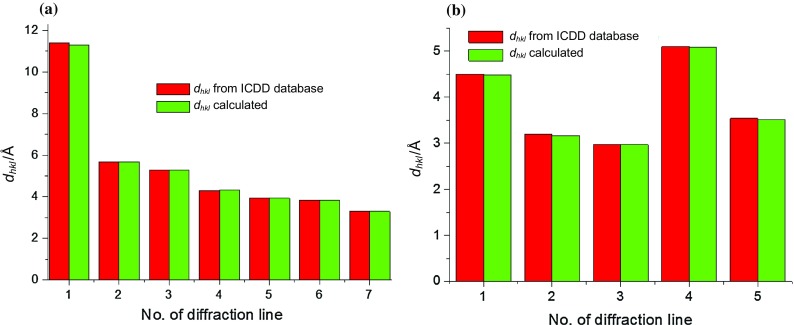
Comparison of the calculated values of *d*_*hkl*_ with the values found in the ICDD Database: **a** Bayer Aspirin, **b** Vitamin C (Apteo)

Below, in Fig. 5, we present an exemplary comparison of the calculated and basic values of *d*_*hkl*_ for Aspirin Bayer and Vitamin C (Apteo). We obtained similar results for the rest of the tested samples. For all samples, the calculated values of *d*_*hkl*_, and those from the ICDD database are in a good agreement, confirming that the investigated samples contain a correct amount of active substance.

The intensities of the diffraction lines depend on the content of the component in the tested preparation. This dependence is not linear, because at the same amount of substance in different mixtures, the intensities of its diffraction lines vary depending on the X-ray absorption coefficient in the mixture. Therefore, different intensity lines for the active substance were observed in the obtained diffraction patterns.

**Fig. 6:**
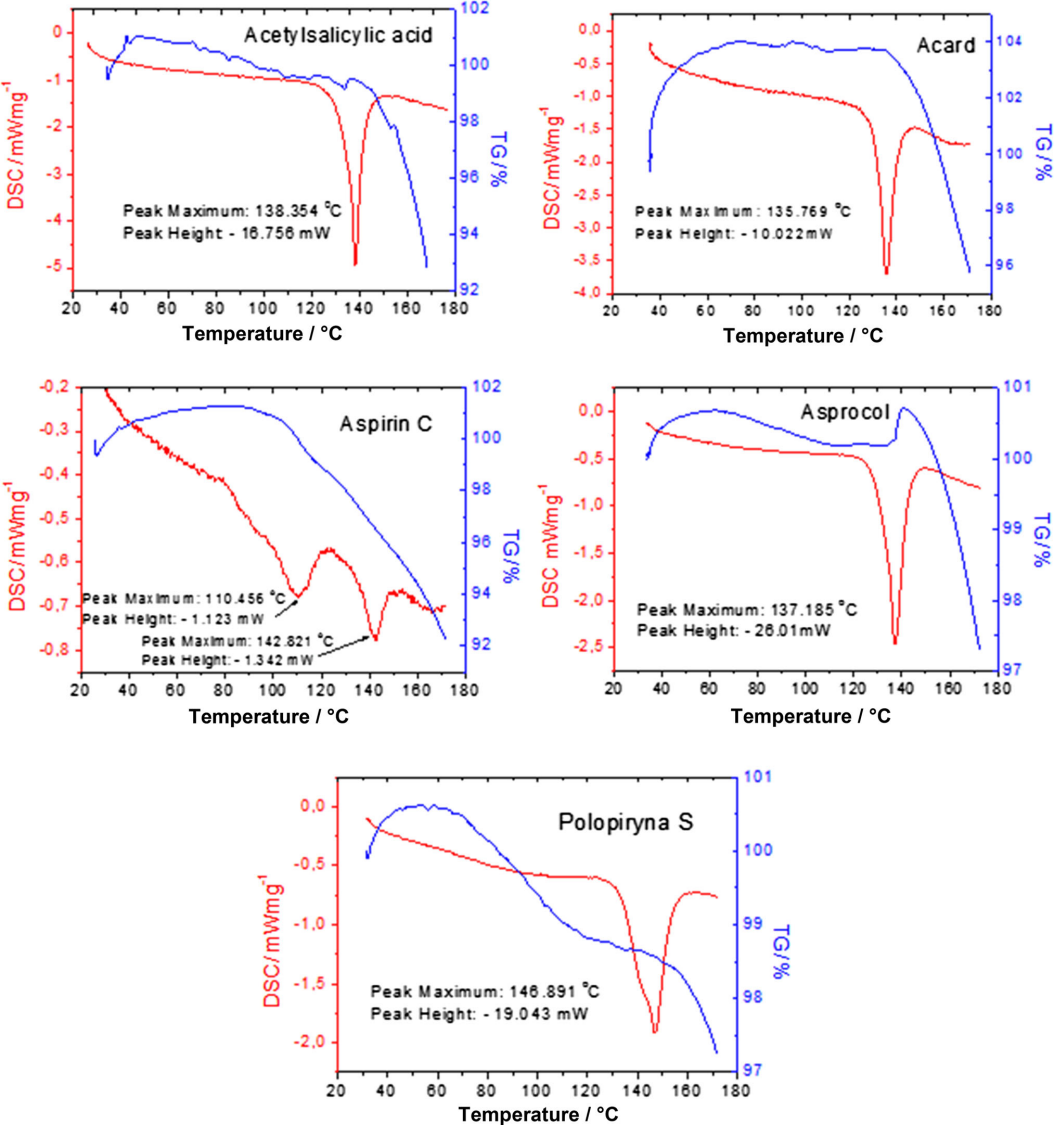
DSC/TG curves for the drugs containing acetylsalicylic acid

The thermal analysis was carried out for drugs containing acetylsalicylic acid. Results of the analysis of DSC and TG curves for the tested drugs are presented in Fig. 6. This analysis showed a weight loss of about 5–10% (depending on the drug), which can indicate a thermal dissociation with a loss of CO_2_ or H_2_O, or emission of gaseous products [11].

During the heating process, we can also observe crystalline transformations. All observed processes (dehydration, melting and crystalline transformation) are accompanied by the endothermic effects in the DSC curves. In Fig. 6, we can observe a good accordance between the value of the melting point and the beginning of the mass loss.

The characteristic endothermic peaks and values of the melting point can be used to confirm the identity of the investigated substances and to evaluate their physicochemical properties. For our investigated samples, all obtained values of melting point are consistent with the commonly available data (Table 5). The melting point for pure acetylsalicylic acid is reported in the temperature range of 134–140 °C [11–14]. It must be noted that in literature we can find slightly higher value of melting point, i.e., 142.6 °C [15]. Only the values for Aspirin C and Polopiryna S are slightly higher. It may be caused by the presence of a different excipient in the composition of this drug. Polopiryna S has cochineal red in its composition (Table 7).

**Table 7:** Obtained values of melting points for the investigated drugs

Drug	Melting point/ °C
Acetylsalicylic acid	138.35
Acard	135.76
Aspirin C	142.82
	110.46
Asprocol	137.18
Polopiryna S	146.89

## Conclusions

Based on the research carried out using the thermoanalytical and X-ray techniques, we can state that a combination of the two methods, XRD and DSC, can be used to distinguish original drugs from counterfeit products, e.g., by checking for the presence of the correct API or by a comparison of the drugs fingerprint. For all investigated samples, the presence of the active substance (acetylsalicylic acid, ascorbic acid) was confirmed. The diffraction lines derived from the other active substances (vitamin C, caffeine, etc.) were identified. In addition to the characteristic lines of the active substances, lines originating from other ingredients have also been identified, i.e., caffeine, paracetamol. The calculated values of the interplanar distance *d*_*hkl*_ are in a good agreement with the data available in the ICDD database.

Determination of the behavior of the investigated drugs at various temperatures allows to define the temperature limits, below which the analyzed substances can be processed without changes in their physicochemical properties. The obtained results may also be useful for an investigation of stability of various drugs and may be used to detect incompatibilities in the drug composition.

## Experimental

### Materials

The selected over-the-counter medicines and dietary supplements:

- Containing acetylsalicylic acid: Acard, Aspirin Bayer, Aspirin C, Aspirin Effect, Coffepirine,
- Containing ascorbic acid: Rutinoscorbin, Vitaminum C Alofarm, Gripex, Pyramidon, Vitaminum C Apteo,
- Pure acetylsalicylic acid and pure ascorbic acid as standards.

### XRPD and DSC procedure

The XRPD measurements were carried out using a Philips PW1050 diffractometer, whereas the DSC measurements were done using a DSC–TG Labsys Evo system. All samples were investigated in a polycrystalline form. Each sample was powdered in an agate mortar.

The X-ray measurements were carried out at room temperature, the angle range 2*θ* = 5°–135°, step 0.02°, *λ*_CuKα_ = 1.5402 Å. Each X-ray measurement was done during 24 h. The DSC measurements were carried out in the flowing air atmosphere with a heating rate of 10 °C/min in the temperature range of 30–190°C.
